# Crystal structure and DFT study of bis­{(*S*)-2-[(2-hy­droxy­benzyl)­amino]-4-methyl­penta­noato-κ^2^ *N*,*O*}(1,10-phenanthroline-κ^2^ *N*,*N*′)nickel(II)

**DOI:** 10.1107/S2056989017012014

**Published:** 2017-08-30

**Authors:** Md. Serajul Haque Faizi, Necmi Dege, Sergey Malinkin

**Affiliations:** a Department of Chemistry, College of Science, Sultan Qaboos University, PO Box 36 Al-Khod 123, Muscat, Sultanate of Oman; b Ondokuz Mayıs University, Arts and Sciences Faculty, Department of Physics, 55139 Atakum–Samsun, Turkey; c Department of Chemistry, National Taras Shevchenko University of Kiev, 64/13, Volodymyrska Street, City of Kyiv, 01601, Ukraine

**Keywords:** crystal structure, nickel(II), phenanthroline, amine, leucine, salicyl­aldehyde, C—H⋯π inter­actions, hydrogen bonding

## Abstract

In the title compound, [Ni(C_13_H_18_NO_3_)_2_(C_12_H_8_N_2_)], the Ni^II^ cation shows a distorted octa­hedral coordination environment. It is formed by two N atoms from the phenanthroline ligand, as well as two N and two O atoms belonging to two 2-[(2-hy­droxy­benz­yl)amino]-4-methyl­penta­noate ligands. Complex mol­ecules are connected into layers propagating along the *ab* plane *via* hydrogen bonds formed by O atoms of carboxyl­ate and phenoxide groups, which are further connected into a three-dimensional motif.

## Abstract 2

The title compound was prepared from an equimolar mixuture of nickel nitrate, phenanthroline and two equivalents of (*S*)-2-(2-hy­droxy­benzyl­amino)-4-methyl­penta­noic acid. The Ni^II^ complex shows a distorted octa­hedral geometry which is stabilized by intra­molecular hydrogen bonds and a weak π–π inter­action.

## Chemical context   

The design and synthesis of metal complexes have attracted considerable attention for their potential applications in catalysis, magnetism, materials science and pharmaceutical chemistry (Che & Siu, 2010 ▸). Mononuclear ethyl­enedi­aminedi­acetate complexes can be used to bind and cleave DNA under physiological conditions (An *et al.*, 2006 ▸) and binuclear complexes containing bipyridyl or phenanthroline units in their structure show anti­viral activity, as well as inhibition of proviral DNA synthesis (Rajendiran *et al.*, 2007 ▸). On the other hand, using bifunctional ligands that are capable of simultaneously coordinating to a metal centre and providing hydrogen bonding gives important experimental data for a better understanding of the key tools in crystal engineering (Burrows, 2004 ▸). Metal complexes of 1,10-phenanthroline (phen) and its derivatives are of increasing inter­est because of their versatile roles in many fields, such as analytical chemistry (Chalk & Tyson, 1994 ▸), catalysis (Samnani *et al.*, 1996 ▸), electrochemical polymerization (Bachas *et al.*, 1997 ▸) and biochemistry (Sammes & Yahioglu, 1994 ▸). 1,10-Phenanthroline is a bidentate chelating ligand with notable coordination ability for transition metal cations. Over the last few decades, the complex formation of transition metal ions with amino acids has also been studied extensively (Auclair *et al.*, 1984 ▸). Amino acid–metallic ion inter­actions are found to be responsible for enzymatic activity and the stability of protein structures (Dinelli *et al.*, 2010 ▸). Nickel is also essential for the healthy life of animals since it is associated with several enzymes (Poellot *et al.*, 1990 ▸) and plays a role in physiological processes as a cofactor in the absorption of iron from the intestine (Nielsen, 1980 ▸). Any change in its concentration leads to metabolic disorder (Kolodziej, 1994 ▸). With the discovery of the biological importance of nickel, it is essential to study its complex formation with amino acids in order to gain a better understanding of the functions of their complexes (Faizi & Sharkina, 2015 ▸). Therefore, we report here the preparation and the crystal structure of a nickel(II) complex with the formula: [Ni(C_13_H_18_NO_3_)_2_(C_12_H_8_N_2_)], (**I**).
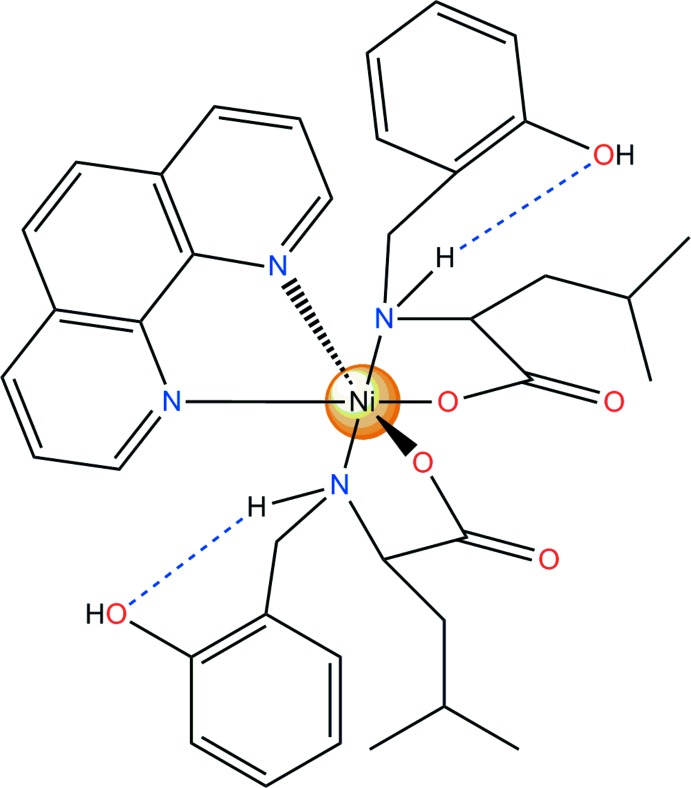


## Structural commentary   

**Figure 1:**
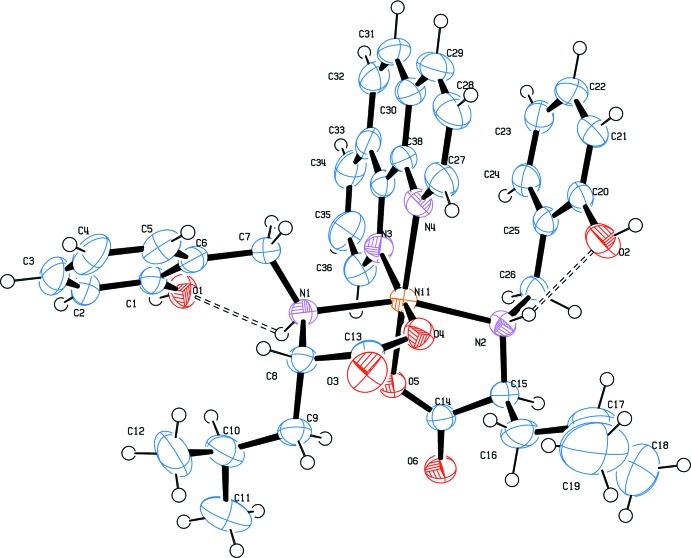
The mol­ecular structure of compound **I**, showing the atom labelling. Displacement ellipsoids are drawn at the 40% probability level.

The complex mol­ecule of **I**, represented in Fig. 1 ▸, contains one crystallographically independent Ni^II^ cation, which is octahedrally coordinated by two mol­ecules of deprotonated 2-[(2-hy­droxy­benz­yl)amino]-4-methyl­penta­noic acid *via* their N atoms and one of the carboxylate atoms each. The coordination environment is com­pleted by one bidentate phenanthroline ligand. The C—O bond lengths in the deprotonated carb­oxy­lic acid groups differ significantly [1.239 (2) and 1.292 (2) Å], which is typical for monodentate carboxyl­ate groups (Wörl *et al.*, 2005*a* ▸,*b* ▸).

The values of the Ni—O bond lengths are similar to those reported in the literature for octa­hedral carboxyl­ate nickel(II) complexes **II–IV** (see §5). However, the corresponding Ni—N separations of 2.101 (3)–2.149 (3) Å are somewhat shorter than found for **III–IV** and similar to that observed in **II**.

Consequently, the slightly distorted octa­hedral coordination is stabilized by intra­molecular N1—H1*A*⋯O1 and N2—H2*A*⋯O2 hydrogen bonds between O atoms of phenoxide moieties and amino groups (Table 1 ▸ and Fig. 1 ▸) and a weak π–π inter­action between the phenanthroline ligand and the phenoxide unit [centroid(N4/C27–C30/C38)⋯centroid(C20–C25) = 3.530 (2) Å].

## Supra­molecular features   

**Figure 2:**
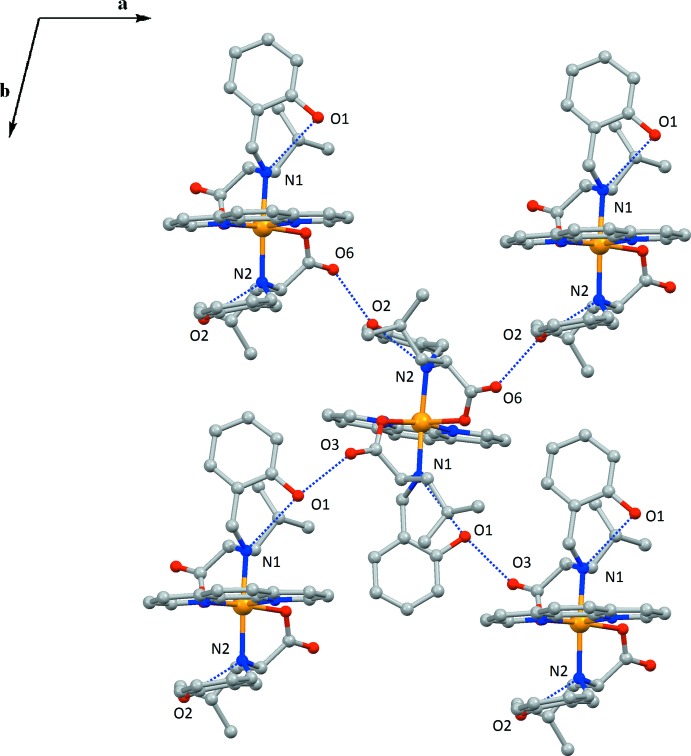
A view of the O—H⋯O hydrogen bonds (dashed lines; see Table 1 ▸) in the crystal of compound **I**, forming layers that are parallel to the *ab* plane. All H atoms have been omitted for clarity.

**Figure 3:**
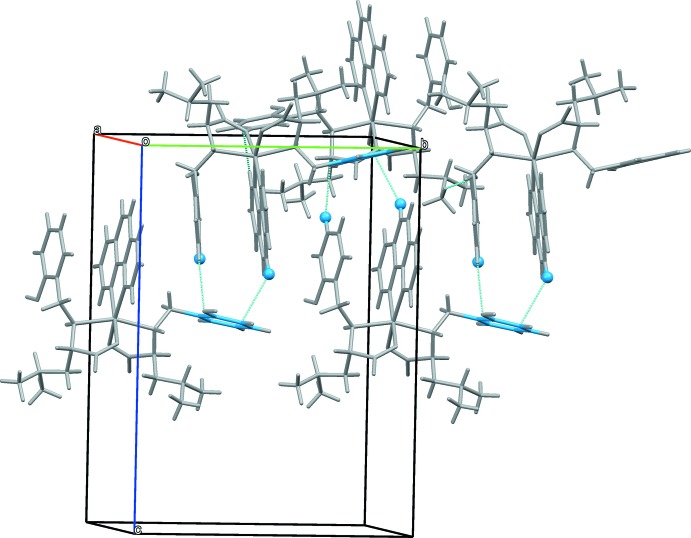
A view along the *b* axis of the crystal packing of compound **I**. The C—H⋯π inter­actions are illustrated by dashed lines. All H atoms have been omitted for clarity.

As shown in Fig. 2 ▸, mol­ecules of **I** are united into layers along the *ab* plane *via* hydrogen bonds formed between the O atoms of carboxyl­ate and phenoxide groups (Table 1 ▸). The layers are stacked *via* weak C—H⋯π inter­actions between the H atoms of phenanthroline ligands and phenoxide moieties [H32⋯centroid(C1–C6) = 3.390 (5) Å and H23⋯centroid(C1–C6) = 3.477 (3) Å] (Fig. 3 ▸).

## DFT study   

The mol­ecular structure used in the theoretical studies of the Ni complex was taken from the X-ray diffraction results, keeping all distances, angles and dihedral angles frozen. Single-point DFT calculations have been carried out using the scalar zeroth-order regular approximation Hamiltonian (Wüllen, 1998 ▸). Single-point ground-state calculations were carried out using the hybrid B3LYP functional as implemented in *ORCA* (Lee *et al.*, 1988 ▸). The present calculation was performed using the additional approximation that the Coulomb integrals are approximated by sum of atom centred *s*, *p*, *d* functions, the auxiliary (or fitting) basis set (Yilmaz *et al.*, 2006 ▸). This allows for efficient treatment of the Coulomb inter­actions and hence reduces calculation times. The Def2-TZVP main and Def2-TZVP/J auxiliary basis sets were used (Pantazis *et al.*, 2008 ▸). The main basis set is of [5*s*3*p*2*d*] quality for Ni, (5*s*2*p*1*d*) for C, N and O, and (2*s*) for H (Weigend & Ahlrichs, 2005 ▸).

**Figure 4:**
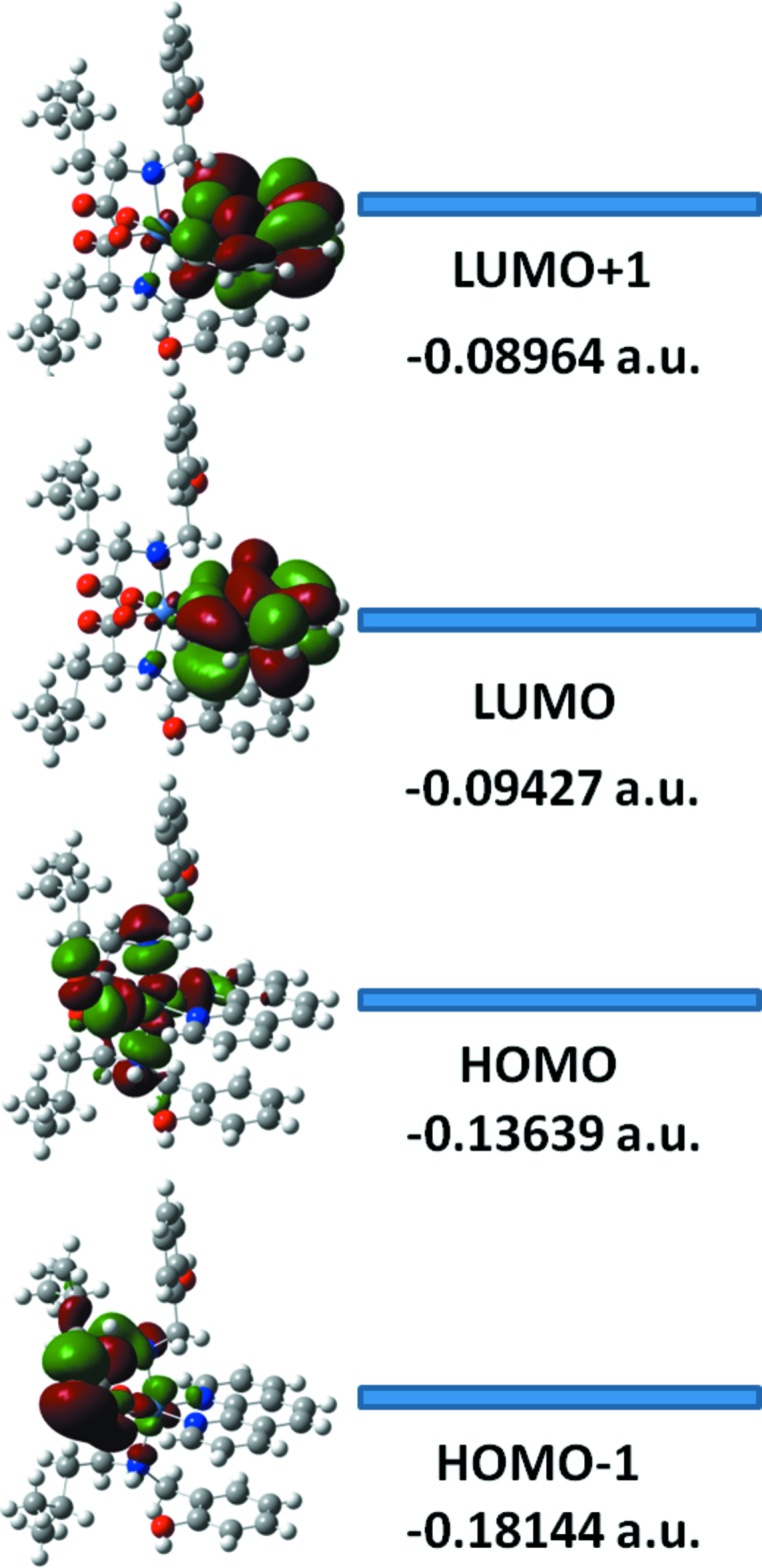
Electron distribution of the HOMO-1, HOMO, LUMO and LUMO+1 energy levels for **I**.

The LUMO and HOMO orbital energy parameters are significantly accountable for the charge transfer, chemical reactivity and kinetic/thermodynamic stability of a mol­ecule. Metal complexes with a small energy gap (Δ*E*) between the HOMO and LUMO are more polarizable, thereby acting as soft mol­ecules with higher chemical reactivity. However, complexes with a large energy gap offer greater stability and low chemical reactivity compared to those with a small HOMO–LUMO energy gap. The DFT study of **I** revealed that the HOMO and HOMO-1 are localized on the N1, N2, O4, O5, O3, O6, C13 and C14 atoms of the amino acid ligand. In addition, the respective mol­ecular orbitals are also partially localized on the Ni^II^ cation, namely in the *d* 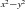 orbital (Fig. 4 ▸). In contrast, LUMO and LUMO+1 are totally delocalized over the phenanthroline moiety. It could therefore be stated that the HOMO and LUMO are mainly composed of σ- and π-type orbitals, respectively, and that intra­molecular charge transfer occurs from the amino acid moiety to the phenanthroline ligand. The HOMO–LUMO gap of **I** was calculated to 0.04212 a.u. and the frontier mol­ecular orbital energies of **I** are also given in Fig. 4 ▸. A comparison of selected geometric data for **I** [Chem scheme1] from calculated (DFT) and X-ray data is given in Table 2 ▸.

## Database survey   

A search of the Cambridge Structural Database (CSD, Version 5.38, update February 2017; Groom *et al.*, 2016 ▸) revealed the structures of three similar compounds, *viz.* (**II**) (IVIKOO; Ji *et al.*, 2011 ▸), (**III**) (FATQAT; Ma *et al.*, 2004 ▸) and (**IV**) (YOWKEA; Skoulika *et al.*, 1995 ▸); all three nickel(II) complexes have similar N_4_O_2_ coordination environments formed by amino­carboxyl­ate and phenanthroline ligands.

## Synthesis and crystallization   

For the preparation of 2-[(2-hy­droxy­benz­yl)amino]-4-methyl­penta­noic acid (HAMA), l-leucine (1.00 g, 6.71 mmol) and LiOH·H_2_O (0.284 g, 6.77 mmol) in anhydrous methanol (30 ml) were stirred for 30 min to dissolve. A methano­lic solution of salicyl­aldehyde (1.44 g, 6.72 mmol) was added dropwise to the above solution. The solution was stirred for 1 h and then treated with sodium borohydride (0.248 g, 6.71 mmol) with constant stirring. The solvent was evaporated and the resulting sticky mass was dissolved in water. A cloudy solution was obtained, which was then acidified with dilute HCl. By maintaining the pH of the solution in the range 5–7 the ligand precipitated as a colourless solid. The solid was filtered off, washed thoroughly with water and finally dried inside a vacuum desiccator (yield 2.08 g, 85%).

For the preparation of the title compound, HAMA (0.500 g, 1.43 mmol) was deprotonated with LiOH·H_2_O (0.060 g, 1.44 mmol) in anhydrous methanol (25 ml), which resulted in a clear colourless solution after 30 min. A methano­lic solution of Ni(NO_3_)_2_·6H_2_O (0.17 g, 0.71 mmol) was added dropwise to the ligand solution with stirring. The colour of the solution changed to green immediately. Phenanthroline (0.13 g, 0.71 mmol) was then added and the reaction mixture was stirred at room temperature for 16 h. The solution was evaporated to dryness with a rotary evaporator. Blue block-shaped crystals, suitable for single-crystal X-ray analysis, were obtained by slow diffusion of diethyl ether into a methano­lic solution of the crude solid over a period of 2–3 d. The crystals were filtered off and washed with diethyl ether (yield 74%).

## Refinement   

Crystal data, data collection and structure refinement details are summarized in Table 3 ▸. The N—H hydrogens were located in a difference Fourier map and refined without constraints. The O—H hydrogens were also located in a difference Fourier map but were constrained to ride on their parent atoms, with *U*_iso_(H) = 1.5*U*_eq_(O). The C-bound H atoms were included in calculated positions and treated as riding atoms, with C—H = 0.95 Å and *U*_iso_(H) = 1.2–1.5*U*_eq_(C).

## Supplementary Material

Crystal structure: contains datablock(s) I. DOI: 10.1107/S2056989017012014/im2482sup1.cif

Structure factors: contains datablock(s) I. DOI: 10.1107/S2056989017012014/im2482Isup2.hkl

CCDC reference: 1548336

Additional supporting information:  crystallographic information; 3D view; checkCIF report

## Figures and Tables

**Table 1:** Hydrogen-bond geometry (Å, °)

*D*—H⋯*A*	*D*—H	H⋯*A*	*D*⋯*A*	*D*—H⋯*A*
O2—H2⋯O6^i^	0.82	1.82	2.597 (5)	158
O1—H1⋯O3^ii^	0.82	1.90	2.686 (5)	161
N2—H2*A*⋯O2	0.98	2.13	2.856 (5)	129
N1—H1*A*⋯O1	0.98	2.45	3.082 (5)	122
C2—H2*B*⋯O3^ii^	0.93	2.56	3.187 (6)	125
C9—H9*A*⋯O5	0.97	2.38	3.212 (6)	143
C16—H16*B*⋯O4	0.97	2.31	3.140 (7)	143
C27—H27⋯O4	0.93	2.58	3.094 (5)	116
C36—H36⋯O5	0.93	2.62	3.132 (6)	115

Symmetry codes: (i) 
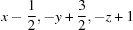
; (ii) 
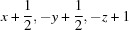
.

**Table 2:** Comparison of selected geometric data for **I** [Chem scheme1] (Å, °) from calculated (DFT) and X-ray data

Bonds	X-ray	B3LYP/6–311G(d,p)
Ni1—N3	2.101 (3)	2.100
Ni1—N4	2.105 (3)	2.105
Ni1—N1	2.141 (3)	2.142
Ni1—N2	2.149 (3)	2.149
Ni1—O5	2.044 (2)	2.044
Ni1—O4	2.051 (3)	2.051
O5—Ni1—O4	101.77 (11)	101.771
N3—Ni1—N2	101.52 (15)	101.510

**Table 3:** Experimental details

Crystal data	
Chemical formula	[Ni(C_13_H_18_NO_3_)_2_(C_12_H_8_N_2_)]
*M*_r_	711.48
Crystal system, space group	Orthorhombic, *P*2_1_2_1_2_1_
Temperature (K)	296
*a*, *b*, *c* (Å)	12.9336 (4), 14.5249 (4), 19.9141 (5)
*V* (Å^3^)	3741.05 (18)
*Z*	4
Radiation type	Mo *K*α
μ (mm^−1^)	0.57
Crystal size (mm)	0.30 × 0.22 × 0.20

Data collection	
Diffractometer	Bruker APEXII CCD area detector
Absorption correction	Multi-scan (*SADABS*; Bruker, 2005 ▸)
*T*_min_, *T*_max_	0.848, 0.895
No. of measured, independent and observed [*I* > 2σ(*I*)] reflections	29587, 8568, 6012
*R*_int_	0.029
(sin θ/λ)_max_ (Å^−1^)	0.650

Refinement	
*R*[*F* ^2^ > 2σ(*F* ^2^)], *wR*(*F* ^2^), *S*	0.043, 0.133, 0.95
No. of reflections	8568
No. of parameters	449
No. of restraints	18
H-atom treatment	H-atom parameters constrained
Δρ_max_, Δρ_min_ (e Å^−3^)	0.76, −0.29
Absolute structure	Refined as an inversion twin
Absolute structure parameter	−0.010 (18)

Computer programs: *APEX2* (Bruker, 2005 ▸), *SAINT* (Bruker, 2005 ▸), *SHELXT2014* (Sheldrick, 2015*a* ▸), *Mercury* (Macrae *et al.*, 2008 ▸), *SHELXTL* (Sheldrick, 2008 ▸), *ORTEP-3 for Windows* (Farrugia, 2012 ▸), *SHELXL2016* (Sheldrick, 2015*b* ▸) and *PLATON* (Spek, 2009 ▸).

## supplementary crystallographic information

### Crystal data

**Table:**

[Ni(C_13_H_18_NO_3_)_2_(C_12_H_8_N_2_)]	*D*_x_ = 1.263 Mg m^−^^3^
*M**_r_* = 711.48	Mo *K*α radiation, λ = 0.71073 Å
Orthorhombic, *P*2_1_2_1_2_1_	Cell parameters from 9936 reflections
*a* = 12.9336 (4) Å	θ = 0.9–0.9°
*b* = 14.5249 (4) Å	µ = 0.57 mm^−^^1^
*c* = 19.9141 (5) Å	*T* = 296 K
*V* = 3741.05 (18) Å^3^	Block, blue
*Z* = 4	0.30 × 0.22 × 0.20 mm
*F*(000) = 1504	

### Data collection

**Table:**

Bruker APEXII CCD area detector diffractometer	8568 independent reflections
Radiation source: fine-focus sealed tube, x-ray	6012 reflections with *I* > 2σ(*I*)
Graphite monochromator	*R*_int_ = 0.029
phi and ω scans	θ_max_ = 27.5°, θ_min_ = 1.7°
Absorption correction: multi-scan multi-scan	*h* = −16→16
*T*_min_ = 0.848, *T*_max_ = 0.895	*k* = −18→18
29587 measured reflections	*l* = −25→25

### Refinement

**Table:**

Refinement on *F*^2^	Hydrogen site location: inferred from neighbouring sites
Least-squares matrix: full	H-atom parameters constrained
*R*[*F*^2^ > 2σ(*F*^2^)] = 0.043	*w* = 1/[σ^2^(*F*_o_^2^) + (0.0876*P*)^2^] where *P* = (*F*_o_^2^ + 2*F*_c_^2^)/3
*wR*(*F*^2^) = 0.133	(Δ/σ)_max_ = 0.001
*S* = 0.95	Δρ_max_ = 0.76 e Å^−^^3^
8568 reflections	Δρ_min_ = −0.29 e Å^−^^3^
449 parameters	Absolute structure: Refined as an inversion twin
18 restraints	Absolute structure parameter: −0.010 (18)

### Special details

**Table:**

Geometry. All esds (except the esd in the dihedral angle between two l.s. planes) are estimated using the full covariance matrix. The cell esds are taken into account individually in the estimation of esds in distances, angles and torsion angles; correlations between esds in cell parameters are only used when they are defined by crystal symmetry. An approximate (isotropic) treatment of cell esds is used for estimating esds involving l.s. planes.
Refinement. Refined as a 2-component inversion twin

### Fractional atomic coordinates and isotropic or equivalent isotropic displacement parameters (Å^2^)

**Table:**

	*x*	*y*	*z*	*U*_iso_*/*U*_eq_	
Ni1	0.52225 (3)	0.50277 (4)	0.46103 (2)	0.04058 (16)	
O5	0.6501 (2)	0.5145 (2)	0.52079 (13)	0.0468 (7)	
O2	0.3621 (3)	0.7541 (3)	0.41899 (14)	0.0724 (10)	
H2	0.311477	0.787276	0.413623	0.109*	
O1	0.6688 (2)	0.2074 (2)	0.43473 (17)	0.0571 (8)	
H1	0.720827	0.175450	0.431692	0.086*	
O4	0.4054 (2)	0.5004 (3)	0.53063 (12)	0.0514 (6)	
O6	0.7370 (2)	0.6064 (2)	0.58909 (15)	0.0616 (9)	
O3	0.3183 (3)	0.4110 (3)	0.60140 (16)	0.0679 (9)	
N4	0.4109 (2)	0.4992 (3)	0.38398 (15)	0.0475 (7)	
N2	0.5284 (3)	0.6495 (2)	0.47445 (15)	0.0419 (8)	
H2A	0.457126	0.670131	0.481619	0.050*	
N3	0.6159 (3)	0.4852 (3)	0.37583 (17)	0.0503 (9)	
N1	0.5230 (3)	0.3595 (2)	0.48649 (15)	0.0435 (8)	
H1A	0.595472	0.341573	0.492509	0.052*	
C38	0.4518 (3)	0.4861 (3)	0.3217 (2)	0.0502 (10)	
C37	0.5622 (4)	0.4787 (3)	0.3174 (2)	0.0483 (10)	
C1	0.5863 (3)	0.1533 (3)	0.45023 (19)	0.0478 (10)	
C20	0.4058 (4)	0.7348 (3)	0.3577 (2)	0.0517 (11)	
C25	0.5103 (4)	0.7109 (3)	0.35645 (19)	0.0482 (10)	
C6	0.4888 (3)	0.1959 (3)	0.45109 (18)	0.0470 (9)	
C14	0.6642 (3)	0.5909 (3)	0.54962 (19)	0.0459 (9)	
C13	0.3911 (3)	0.4256 (3)	0.5619 (2)	0.0469 (10)	
C2	0.5965 (4)	0.0606 (3)	0.4652 (2)	0.0603 (12)	
H2B	0.661691	0.033535	0.465867	0.072*	
C33	0.6104 (5)	0.4661 (3)	0.2544 (2)	0.0616 (13)	
C7	0.4781 (3)	0.2962 (3)	0.4355 (2)	0.0482 (9)	
H7A	0.511075	0.308224	0.392633	0.058*	
H7B	0.405176	0.310368	0.430582	0.058*	
C9	0.5481 (4)	0.3555 (4)	0.6112 (2)	0.0568 (12)	
H9A	0.593990	0.407310	0.603534	0.068*	
H9B	0.509724	0.367962	0.652028	0.068*	
C30	0.3934 (4)	0.4825 (3)	0.2632 (2)	0.0652 (14)	
C8	0.4719 (3)	0.3501 (3)	0.55296 (18)	0.0464 (10)	
H8	0.436947	0.290295	0.554666	0.056*	
C15	0.5844 (3)	0.6659 (3)	0.5380 (2)	0.0488 (9)	
H15	0.620696	0.724908	0.534388	0.059*	
C21	0.3505 (4)	0.7395 (3)	0.2990 (2)	0.0633 (12)	
H21	0.280440	0.753963	0.300120	0.076*	
C24	0.5575 (4)	0.6950 (3)	0.2947 (2)	0.0569 (11)	
H24	0.627329	0.679860	0.293289	0.068*	
C5	0.4047 (4)	0.1405 (4)	0.4663 (2)	0.0647 (13)	
H5	0.339253	0.166923	0.467603	0.078*	
C16	0.5130 (5)	0.6701 (4)	0.5987 (2)	0.0756 (15)	
H16A	0.554693	0.668247	0.639188	0.091*	
H16B	0.469059	0.616033	0.598668	0.091*	
C4	0.4145 (5)	0.0476 (4)	0.4798 (3)	0.0767 (17)	
H4	0.356250	0.012325	0.489077	0.092*	
C23	0.5026 (5)	0.7013 (3)	0.2357 (2)	0.0642 (13)	
H23	0.535243	0.691361	0.194712	0.077*	
C22	0.3988 (5)	0.7226 (4)	0.2379 (2)	0.0678 (14)	
H22	0.360883	0.725703	0.198237	0.081*	
C32	0.5485 (6)	0.4609 (4)	0.1963 (3)	0.0774 (16)	
H32	0.579803	0.451734	0.154833	0.093*	
C26	0.5726 (4)	0.7091 (3)	0.4201 (2)	0.0526 (10)	
H26A	0.641737	0.687488	0.409610	0.063*	
H26B	0.578654	0.771477	0.436947	0.063*	
C34	0.7167 (5)	0.4601 (4)	0.2532 (3)	0.0755 (16)	
H34	0.751273	0.451436	0.212747	0.091*	
C28	0.2451 (4)	0.5091 (4)	0.3321 (3)	0.0772 (15)	
H28	0.174436	0.518730	0.337209	0.093*	
C31	0.4447 (7)	0.4688 (4)	0.1996 (2)	0.086 (2)	
H31	0.405790	0.465412	0.160442	0.104*	
C3	0.5097 (5)	0.0080 (4)	0.4792 (2)	0.0736 (14)	
H3	0.516635	−0.054468	0.488318	0.088*	
C29	0.2855 (5)	0.4945 (5)	0.2700 (3)	0.0809 (16)	
H29	0.242723	0.492516	0.232541	0.097*	
C27	0.3098 (3)	0.5095 (4)	0.3884 (2)	0.0614 (12)	
H27	0.280372	0.517393	0.430589	0.074*	
C36	0.7183 (4)	0.4780 (4)	0.3723 (3)	0.0621 (13)	
H36	0.756573	0.480433	0.411765	0.075*	
C10	0.6139 (4)	0.2694 (4)	0.6222 (3)	0.0733 (15)	
H10	0.643633	0.252149	0.578763	0.088*	
C35	0.7705 (5)	0.4669 (4)	0.3114 (3)	0.0821 (17)	
H35	0.842317	0.464102	0.310735	0.098*	
C11	0.7018 (5)	0.2873 (6)	0.6694 (3)	0.105 (2)	
H11A	0.741147	0.339042	0.653607	0.157*	
H11B	0.745548	0.233966	0.671362	0.157*	
H11C	0.675180	0.300390	0.713330	0.157*	
C12	0.5484 (7)	0.1884 (5)	0.6466 (4)	0.118 (3)	
H12A	0.531973	0.196611	0.693223	0.177*	
H12B	0.586403	0.132159	0.640902	0.177*	
H12C	0.485621	0.185509	0.620937	0.177*	
C17	0.4436 (7)	0.7575 (8)	0.6008 (3)	0.127 (2)	
H17	0.417295	0.771165	0.555691	0.153*	
C19	0.3515 (8)	0.7320 (10)	0.6487 (5)	0.198 (5)	
H19A	0.345722	0.666232	0.651790	0.297*	
H19B	0.288408	0.756982	0.631046	0.297*	
H19C	0.364186	0.757147	0.692486	0.297*	
C18	0.5090 (12)	0.8401 (8)	0.6272 (5)	0.193 (4)	
H18A	0.532140	0.827174	0.672069	0.290*	
H18B	0.467315	0.894785	0.627429	0.290*	
H18C	0.567741	0.849136	0.598544	0.290*	

### Atomic displacement parameters (Å^2^)

**Table:**

	*U*^11^	*U*^22^	*U*^33^	*U*^12^	*U*^13^	*U*^23^
Ni1	0.0418 (2)	0.0431 (3)	0.0368 (2)	0.0001 (3)	−0.00271 (18)	0.0003 (2)
O5	0.0454 (13)	0.0433 (19)	0.0517 (14)	−0.0012 (14)	−0.0096 (11)	−0.0007 (13)
O2	0.082 (2)	0.090 (3)	0.0458 (17)	0.040 (2)	0.0103 (15)	0.0084 (16)
O1	0.0533 (16)	0.0481 (19)	0.0699 (19)	−0.0028 (15)	−0.0016 (15)	0.0030 (15)
O4	0.0505 (14)	0.0540 (18)	0.0497 (14)	0.0091 (18)	0.0030 (11)	−0.0002 (18)
O6	0.0625 (18)	0.056 (2)	0.0662 (19)	−0.0065 (15)	−0.0276 (16)	−0.0032 (15)
O3	0.0606 (18)	0.074 (2)	0.070 (2)	−0.0105 (17)	0.0182 (17)	−0.0040 (17)
N4	0.0471 (16)	0.0445 (19)	0.0510 (17)	−0.001 (2)	−0.0075 (13)	0.0064 (19)
N2	0.0456 (18)	0.0416 (19)	0.0386 (16)	0.0022 (16)	−0.0017 (15)	0.0042 (13)
N3	0.0548 (19)	0.050 (3)	0.0459 (17)	0.0001 (18)	0.0046 (14)	0.0006 (17)
N1	0.0477 (19)	0.045 (2)	0.0383 (16)	−0.0025 (17)	−0.0040 (15)	−0.0011 (13)
C38	0.072 (3)	0.037 (3)	0.043 (2)	−0.004 (2)	−0.0118 (17)	0.0045 (17)
C37	0.070 (2)	0.034 (3)	0.041 (2)	−0.0006 (19)	0.0002 (19)	0.0013 (16)
C1	0.061 (2)	0.042 (2)	0.041 (2)	−0.007 (2)	−0.0039 (18)	0.0013 (17)
C20	0.069 (3)	0.042 (3)	0.044 (2)	0.013 (2)	0.003 (2)	0.0062 (18)
C25	0.066 (3)	0.036 (2)	0.042 (2)	0.002 (2)	0.0048 (19)	0.0047 (16)
C6	0.055 (2)	0.046 (2)	0.040 (2)	−0.005 (2)	−0.0035 (18)	−0.0070 (17)
C14	0.051 (2)	0.046 (3)	0.041 (2)	−0.0065 (19)	−0.0032 (17)	0.0009 (18)
C13	0.044 (2)	0.056 (3)	0.040 (2)	−0.009 (2)	−0.0013 (17)	−0.0047 (19)
C2	0.082 (3)	0.044 (3)	0.055 (3)	−0.003 (2)	−0.004 (2)	−0.002 (2)
C33	0.101 (4)	0.037 (3)	0.047 (2)	0.000 (2)	0.007 (3)	−0.0041 (18)
C7	0.054 (2)	0.047 (3)	0.043 (2)	0.003 (2)	−0.0104 (19)	−0.0044 (17)
C9	0.074 (3)	0.059 (3)	0.038 (2)	−0.006 (2)	−0.003 (2)	−0.0003 (19)
C30	0.095 (4)	0.045 (3)	0.055 (3)	−0.011 (3)	−0.025 (2)	0.006 (2)
C8	0.055 (2)	0.046 (3)	0.038 (2)	−0.0071 (19)	−0.0025 (17)	0.0020 (16)
C15	0.058 (2)	0.046 (2)	0.043 (2)	−0.0005 (19)	−0.008 (2)	−0.0009 (18)
C21	0.077 (3)	0.058 (3)	0.055 (3)	0.011 (3)	−0.008 (2)	0.009 (2)
C24	0.072 (3)	0.050 (3)	0.049 (2)	0.004 (2)	0.014 (2)	0.008 (2)
C5	0.065 (3)	0.072 (4)	0.057 (3)	−0.016 (3)	0.008 (2)	−0.016 (3)
C16	0.083 (3)	0.099 (4)	0.044 (2)	0.026 (3)	−0.003 (2)	−0.016 (2)
C4	0.097 (4)	0.062 (4)	0.071 (3)	−0.035 (3)	0.021 (3)	−0.015 (3)
C23	0.097 (4)	0.052 (3)	0.044 (2)	0.008 (3)	0.012 (2)	0.0053 (19)
C22	0.100 (4)	0.062 (3)	0.041 (2)	0.001 (3)	−0.011 (2)	0.007 (2)
C32	0.126 (5)	0.058 (3)	0.048 (3)	0.009 (3)	0.000 (3)	−0.007 (2)
C26	0.059 (2)	0.047 (3)	0.051 (2)	−0.001 (2)	0.004 (2)	0.0077 (19)
C34	0.104 (4)	0.061 (3)	0.061 (3)	−0.001 (3)	0.033 (3)	−0.008 (2)
C28	0.057 (2)	0.072 (4)	0.103 (4)	−0.010 (3)	−0.031 (3)	0.015 (4)
C31	0.159 (6)	0.060 (4)	0.040 (3)	−0.002 (4)	−0.029 (3)	−0.003 (2)
C3	0.119 (4)	0.043 (3)	0.059 (3)	−0.021 (4)	0.014 (3)	0.001 (2)
C29	0.096 (4)	0.068 (4)	0.078 (3)	−0.013 (4)	−0.044 (3)	0.013 (3)
C27	0.057 (2)	0.058 (3)	0.069 (3)	−0.005 (3)	−0.006 (2)	0.014 (3)
C36	0.055 (2)	0.068 (4)	0.063 (3)	0.004 (2)	0.010 (2)	−0.001 (2)
C10	0.073 (3)	0.088 (4)	0.059 (3)	0.015 (3)	−0.012 (3)	0.011 (3)
C35	0.074 (3)	0.085 (5)	0.087 (4)	0.000 (3)	0.026 (3)	−0.019 (3)
C11	0.084 (4)	0.155 (7)	0.075 (4)	0.013 (4)	−0.019 (3)	0.025 (4)
C12	0.150 (7)	0.074 (5)	0.129 (6)	−0.005 (5)	−0.048 (5)	0.043 (4)
C17	0.133 (5)	0.179 (6)	0.069 (3)	0.056 (4)	−0.008 (3)	−0.025 (4)
C19	0.143 (7)	0.323 (12)	0.128 (6)	0.110 (8)	0.014 (6)	−0.057 (8)
C18	0.293 (11)	0.140 (8)	0.147 (7)	0.083 (8)	−0.016 (9)	−0.040 (6)

### Geometric parameters (Å, º)

**Table:**

Ni1—O5	2.044 (2)	C15—C16	1.522 (7)
Ni1—O4	2.051 (3)	C15—H15	0.9800
Ni1—N3	2.101 (3)	C21—C22	1.390 (7)
Ni1—N4	2.105 (3)	C21—H21	0.9300
Ni1—N1	2.141 (3)	C24—C23	1.376 (7)
Ni1—N2	2.149 (3)	C24—H24	0.9300
O5—C14	1.262 (5)	C5—C4	1.382 (8)
O2—C20	1.373 (5)	C5—H5	0.9300
O2—H2	0.8200	C16—C17	1.555 (10)
O1—C1	1.360 (5)	C16—H16A	0.9700
O1—H1	0.8200	C16—H16B	0.9700
O4—C13	1.265 (6)	C4—C3	1.359 (8)
O6—C14	1.247 (5)	C4—H4	0.9300
O3—C13	1.245 (5)	C23—C22	1.379 (8)
N4—C27	1.318 (5)	C23—H23	0.9300
N4—C38	1.362 (5)	C22—H22	0.9300
N2—C15	1.477 (5)	C32—C31	1.348 (9)
N2—C26	1.500 (5)	C32—H32	0.9300
N2—H2A	0.9800	C26—H26A	0.9700
N3—C36	1.330 (6)	C26—H26B	0.9700
N3—C37	1.359 (5)	C34—C35	1.355 (9)
N1—C8	1.486 (5)	C34—H34	0.9300
N1—C7	1.489 (5)	C28—C29	1.359 (8)
N1—H1A	0.9800	C28—C27	1.398 (6)
C38—C30	1.389 (6)	C28—H28	0.9300
C38—C37	1.433 (6)	C31—H31	0.9300
C37—C33	1.414 (6)	C3—H3	0.9300
C1—C2	1.386 (6)	C29—H29	0.9300
C1—C6	1.405 (6)	C27—H27	0.9300
C20—C21	1.373 (6)	C36—C35	1.397 (7)
C20—C25	1.395 (7)	C36—H36	0.9300
C25—C24	1.392 (6)	C10—C11	1.497 (8)
C25—C26	1.502 (6)	C10—C12	1.529 (9)
C6—C5	1.386 (6)	C10—H10	0.9800
C6—C7	1.496 (6)	C35—H35	0.9300
C14—C15	1.519 (6)	C11—H11A	0.9600
C13—C8	1.525 (6)	C11—H11B	0.9600
C2—C3	1.386 (7)	C11—H11C	0.9600
C2—H2B	0.9300	C12—H12A	0.9600
C33—C34	1.377 (9)	C12—H12B	0.9600
C33—C32	1.408 (8)	C12—H12C	0.9600
C7—H7A	0.9700	C17—C19	1.570 (14)
C7—H7B	0.9700	C17—C18	1.560 (15)
C9—C10	1.529 (8)	C17—H17	0.9800
C9—C8	1.524 (6)	C19—H19A	0.9600
C9—H9A	0.9700	C19—H19B	0.9600
C9—H9B	0.9700	C19—H19C	0.9600
C30—C29	1.413 (8)	C18—H18A	0.9600
C30—C31	1.444 (8)	C18—H18B	0.9600
C8—H8	0.9800	C18—H18C	0.9600
			
O5—Ni1—O4	101.77 (11)	C20—C21—C22	120.2 (5)
O5—Ni1—N3	90.80 (12)	C20—C21—H21	119.9
O4—Ni1—N3	165.66 (13)	C22—C21—H21	119.9
O5—Ni1—N4	168.45 (12)	C23—C24—C25	121.1 (4)
O4—Ni1—N4	89.30 (11)	C23—C24—H24	119.4
N3—Ni1—N4	78.64 (13)	C25—C24—H24	119.4
O5—Ni1—N1	86.54 (13)	C4—C5—C6	122.5 (5)
O4—Ni1—N1	80.03 (14)	C4—C5—H5	118.7
N3—Ni1—N1	94.04 (15)	C6—C5—H5	118.7
N4—Ni1—N1	98.73 (16)	C15—C16—C17	113.8 (5)
O5—Ni1—N2	79.32 (13)	C15—C16—H16A	108.8
O4—Ni1—N2	87.72 (14)	C17—C16—H16A	108.8
N3—Ni1—N2	101.52 (15)	C15—C16—H16B	108.8
N4—Ni1—N2	98.07 (16)	C17—C16—H16B	108.8
N1—Ni1—N2	159.02 (12)	H16A—C16—H16B	107.7
C14—O5—Ni1	117.1 (3)	C3—C4—C5	119.6 (5)
C20—O2—H2	109.5	C3—C4—H4	120.2
C1—O1—H1	109.5	C5—C4—H4	120.2
C13—O4—Ni1	117.1 (3)	C22—C23—C24	119.4 (4)
C27—N4—C38	117.5 (3)	C22—C23—H23	120.3
C27—N4—Ni1	128.8 (3)	C24—C23—H23	120.3
C38—N4—Ni1	113.6 (2)	C23—C22—C21	120.3 (4)
C15—N2—C26	109.8 (3)	C23—C22—H22	119.8
C15—N2—Ni1	106.5 (2)	C21—C22—H22	119.8
C26—N2—Ni1	119.8 (3)	C31—C32—C33	121.5 (5)
C15—N2—H2A	106.7	C31—C32—H32	119.3
C26—N2—H2A	106.7	C33—C32—H32	119.3
Ni1—N2—H2A	106.7	N2—C26—C25	114.5 (4)
C36—N3—C37	117.3 (4)	N2—C26—H26A	108.6
C36—N3—Ni1	128.8 (3)	C25—C26—H26A	108.6
C37—N3—Ni1	113.9 (3)	N2—C26—H26B	108.6
C8—N1—C7	112.1 (3)	C25—C26—H26B	108.6
C8—N1—Ni1	107.4 (2)	H26A—C26—H26B	107.6
C7—N1—Ni1	115.9 (2)	C35—C34—C33	119.6 (5)
C8—N1—H1A	107.0	C35—C34—H34	120.2
C7—N1—H1A	107.0	C33—C34—H34	120.2
Ni1—N1—H1A	107.0	C29—C28—C27	119.9 (5)
N4—C38—C30	123.9 (4)	C29—C28—H28	120.0
N4—C38—C37	116.9 (3)	C27—C28—H28	120.0
C30—C38—C37	119.2 (4)	C32—C31—C30	120.8 (5)
N3—C37—C33	122.9 (4)	C32—C31—H31	119.6
N3—C37—C38	117.0 (4)	C30—C31—H31	119.6
C33—C37—C38	120.1 (4)	C4—C3—C2	120.2 (5)
O1—C1—C2	122.4 (4)	C4—C3—H3	119.9
O1—C1—C6	116.9 (4)	C2—C3—H3	119.9
C2—C1—C6	120.7 (4)	C28—C29—C30	119.1 (4)
O2—C20—C21	122.1 (4)	C28—C29—H29	120.4
O2—C20—C25	117.8 (4)	C30—C29—H29	120.4
C21—C20—C25	120.1 (4)	N4—C27—C28	122.7 (5)
C20—C25—C24	118.8 (4)	N4—C27—H27	118.7
C20—C25—C26	120.6 (4)	C28—C27—H27	118.7
C24—C25—C26	120.5 (4)	N3—C36—C35	122.4 (5)
C5—C6—C1	116.8 (4)	N3—C36—H36	118.8
C5—C6—C7	122.6 (4)	C35—C36—H36	118.8
C1—C6—C7	120.6 (4)	C11—C10—C12	110.8 (5)
O6—C14—O5	123.7 (4)	C11—C10—C9	111.8 (5)
O6—C14—C15	118.6 (4)	C12—C10—C9	111.5 (5)
O5—C14—C15	117.6 (3)	C11—C10—H10	107.5
O3—C13—O4	124.6 (4)	C12—C10—H10	107.5
O3—C13—C8	118.0 (4)	C9—C10—H10	107.5
O4—C13—C8	117.3 (3)	C34—C35—C36	120.2 (5)
C3—C2—C1	120.1 (5)	C34—C35—H35	119.9
C3—C2—H2B	120.0	C36—C35—H35	119.9
C1—C2—H2B	120.0	C10—C11—H11A	109.5
C34—C33—C32	123.4 (5)	C10—C11—H11B	109.5
C34—C33—C37	117.6 (5)	H11A—C11—H11B	109.5
C32—C33—C37	118.9 (5)	C10—C11—H11C	109.5
N1—C7—C6	115.1 (3)	H11A—C11—H11C	109.5
N1—C7—H7A	108.5	H11B—C11—H11C	109.5
C6—C7—H7A	108.5	C10—C12—H12A	109.5
N1—C7—H7B	108.5	C10—C12—H12B	109.5
C6—C7—H7B	108.5	H12A—C12—H12B	109.5
H7A—C7—H7B	107.5	C10—C12—H12C	109.5
C10—C9—C8	115.3 (4)	H12A—C12—H12C	109.5
C10—C9—H9A	108.4	H12B—C12—H12C	109.5
C8—C9—H9A	108.4	C19—C17—C16	105.2 (8)
C10—C9—H9B	108.4	C19—C17—C18	112.8 (8)
C8—C9—H9B	108.4	C16—C17—C18	108.8 (7)
H9A—C9—H9B	107.5	C19—C17—H17	110.0
C38—C30—C29	116.9 (5)	C16—C17—H17	110.0
C38—C30—C31	119.4 (5)	C18—C17—H17	110.0
C29—C30—C31	123.7 (5)	C17—C19—H19A	109.5
N1—C8—C13	110.0 (3)	C17—C19—H19B	109.5
N1—C8—C9	112.6 (3)	H19A—C19—H19B	109.5
C13—C8—C9	108.5 (3)	C17—C19—H19C	109.5
N1—C8—H8	108.5	H19A—C19—H19C	109.5
C13—C8—H8	108.5	H19B—C19—H19C	109.5
C9—C8—H8	108.5	C17—C18—H18A	109.5
N2—C15—C16	112.9 (4)	C17—C18—H18B	109.5
N2—C15—C14	110.4 (3)	H18A—C18—H18B	109.5
C16—C15—C14	108.7 (4)	C17—C18—H18C	109.5
N2—C15—H15	108.3	H18A—C18—H18C	109.5
C16—C15—H15	108.3	H18B—C18—H18C	109.5
C14—C15—H15	108.3		
			
C27—N4—C38—C30	−0.5 (7)	C26—N2—C15—C16	138.5 (4)
Ni1—N4—C38—C30	−179.2 (4)	Ni1—N2—C15—C16	−90.4 (4)
C27—N4—C38—C37	177.6 (5)	C26—N2—C15—C14	−99.6 (4)
Ni1—N4—C38—C37	−1.2 (5)	Ni1—N2—C15—C14	31.5 (4)
C36—N3—C37—C33	−0.8 (7)	O6—C14—C15—N2	159.8 (3)
Ni1—N3—C37—C33	−179.5 (3)	O5—C14—C15—N2	−23.3 (5)
C36—N3—C37—C38	179.7 (4)	O6—C14—C15—C16	−75.8 (5)
Ni1—N3—C37—C38	0.9 (5)	O5—C14—C15—C16	101.1 (5)
N4—C38—C37—N3	0.2 (7)	O2—C20—C21—C22	177.6 (5)
C30—C38—C37—N3	178.3 (4)	C25—C20—C21—C22	−1.6 (7)
N4—C38—C37—C33	−179.4 (4)	C20—C25—C24—C23	−0.9 (6)
C30—C38—C37—C33	−1.2 (7)	C26—C25—C24—C23	−176.4 (4)
O2—C20—C25—C24	−177.1 (4)	C1—C6—C5—C4	−0.4 (6)
C21—C20—C25—C24	2.1 (7)	C7—C6—C5—C4	179.0 (4)
O2—C20—C25—C26	−1.6 (6)	N2—C15—C16—C17	−70.1 (6)
C21—C20—C25—C26	177.6 (4)	C14—C15—C16—C17	167.1 (5)
O1—C1—C6—C5	179.5 (4)	C6—C5—C4—C3	1.1 (7)
C2—C1—C6—C5	−1.1 (6)	C25—C24—C23—C22	−0.8 (7)
O1—C1—C6—C7	0.0 (5)	C24—C23—C22—C21	1.4 (8)
C2—C1—C6—C7	179.5 (4)	C20—C21—C22—C23	−0.2 (8)
Ni1—O5—C14—O6	177.6 (3)	C34—C33—C32—C31	−178.7 (5)
Ni1—O5—C14—C15	0.9 (5)	C37—C33—C32—C31	0.8 (8)
Ni1—O4—C13—O3	−173.8 (3)	C15—N2—C26—C25	−169.7 (3)
Ni1—O4—C13—C8	9.4 (4)	Ni1—N2—C26—C25	66.6 (4)
O1—C1—C2—C3	−178.6 (4)	C20—C25—C26—N2	55.0 (6)
C6—C1—C2—C3	2.0 (7)	C24—C25—C26—N2	−129.5 (4)
N3—C37—C33—C34	0.0 (7)	C32—C33—C34—C35	179.2 (5)
C38—C37—C33—C34	179.6 (5)	C37—C33—C34—C35	−0.3 (7)
N3—C37—C33—C32	−179.5 (4)	C33—C32—C31—C30	−0.3 (8)
C38—C37—C33—C32	0.0 (7)	C38—C30—C31—C32	−0.9 (8)
C8—N1—C7—C6	−62.0 (5)	C29—C30—C31—C32	177.7 (6)
Ni1—N1—C7—C6	174.2 (3)	C5—C4—C3—C2	−0.1 (8)
C5—C6—C7—N1	112.1 (4)	C1—C2—C3—C4	−1.4 (7)
C1—C6—C7—N1	−68.5 (5)	C27—C28—C29—C30	−1.7 (9)
N4—C38—C30—C29	0.9 (7)	C38—C30—C29—C28	0.2 (8)
C37—C38—C30—C29	−177.1 (5)	C31—C30—C29—C28	−178.5 (5)
N4—C38—C30—C31	179.7 (5)	C38—N4—C27—C28	−1.1 (8)
C37—C38—C30—C31	1.7 (7)	Ni1—N4—C27—C28	177.4 (4)
C7—N1—C8—C13	−98.4 (4)	C29—C28—C27—N4	2.3 (9)
Ni1—N1—C8—C13	30.0 (4)	C37—N3—C36—C35	1.8 (8)
C7—N1—C8—C9	140.4 (4)	Ni1—N3—C36—C35	−179.7 (4)
Ni1—N1—C8—C9	−91.2 (4)	C8—C9—C10—C11	167.2 (4)
O3—C13—C8—N1	155.4 (4)	C8—C9—C10—C12	−68.1 (6)
O4—C13—C8—N1	−27.6 (5)	C33—C34—C35—C36	1.3 (8)
O3—C13—C8—C9	−80.9 (5)	N3—C36—C35—C34	−2.1 (9)
O4—C13—C8—C9	96.1 (4)	C15—C16—C17—C19	159.5 (6)
C10—C9—C8—N1	−75.5 (5)	C15—C16—C17—C18	−79.4 (7)
C10—C9—C8—C13	162.4 (4)		

### Hydrogen-bond geometry (Å, º)

**Table:**

*D*—H···*A*	*D*—H	H···*A*	*D*···*A*	*D*—H···*A*
O2—H2···O6^i^	0.82	1.82	2.597 (5)	158
O1—H1···O3^ii^	0.82	1.90	2.686 (5)	161
N2—H2*A*···O2	0.98	2.13	2.856 (5)	129
N1—H1*A*···O1	0.98	2.45	3.082 (5)	122
C2—H2*B*···O3^ii^	0.93	2.56	3.187 (6)	125
C9—H9*A*···O5	0.97	2.38	3.212 (6)	143
C16—H16*B*···O4	0.97	2.31	3.140 (7)	143
C27—H27···O4	0.93	2.58	3.094 (5)	116
C36—H36···O5	0.93	2.62	3.132 (6)	115

Symmetry codes: (i) *x*−1/2, −*y*+3/2, −*z*+1; (ii) *x*+1/2, −*y*+1/2, −*z*+1.

## References

[bb1] An, Y., Liu, S. D., Deng, S. Y., Ji, L. N. & Mao, Z. W. J. (2006). *J. Inorg. Biochem.* **100**, 1586–1593. 10.1016/j.jinorgbio.2006.05.002 16844224

[bb2] Auclair, C., Voisin, E., Banoun, H., Paoletti, C., Bernadou, J. & Meunier, B. (1984). *J. Med. Chem.* **27**, 1161–1166. 10.1021/jm00375a013 6471070

[bb3] Bachas, L. G., Cullen, L., Hutchins, R. S. & Scott, D. L. (1997). *J. Chem. Soc. Dalton Trans.* pp. 1571–1578.

[bb4] Bruker (2005). *APEX2*, *SAINT* and *SADABS*. Bruker AXS Inc., Madison, Wisconsin, USA.

[bb5] Burrows, A. D. (2004). *Struct. Bond.* **108**, 55–96.

[bb6] Chalk, S. J. & Tyson, J. F. (1994). *Anal. Chem.* **66**, 660–666.

[bb7] Che, C. M. & Siu, F. M. (2010). *Curr. Opin. Chem. Biol.* **14**, 255–261. 10.1016/j.cbpa.2009.11.015 20018553

[bb8] Dinelli, L. R., Bezerra, T. M. & Sene, J. J. (2010). *Curr. Res. Chem*, **2**, 18–23.

[bb9] Faizi, M. S. H. & Sharkina, N. O. (2015). *Acta Cryst.* E**71**, 195–198. 10.1107/S2056989015001085 PMC4384555 25878817

[bb10] Farrugia, L. J. (2012). *J. Appl. Cryst.* **45**, 849–854.

[bb11] Groom, C. R., Bruno, I. J., Lightfoot, M. P. & Ward, S. C. (2016). *Acta Cryst.* B**72**, 171–179. 10.1107/S2052520616003954 PMC4822653 27048719

[bb12] Ji, J.-L., Huang, L.-Q., Cai, Y., Yu, L.-J. & Zhou, Z.-H. (2011). *J. Mol. Struct.* **994**, 70–74.

[bb13] Kolodziej, A. F. (1994). *Progress in Inorganic Chemistry*, Vol. 41, edited by K. D. Karlin, pp. 493–523. New York: Wiley.

[bb14] Lee, C., Yang, W. & Parr, R. G. (1988). *Phys. Rev. B*, **37**, 785–789. 10.1103/physrevb.37.785 9944570

[bb15] Ma, L.-F., Liang, F.-P., Qin, H.-C., Hu, R.-X. & Zhang, M.-B. (2004). *Chin. J. Struct. Chem. (Jiegou Huaxue)*, **23**, 1376.

[bb16] Macrae, C. F., Bruno, I. J., Chisholm, J. A., Edgington, P. R., McCabe, P., Pidcock, E., Rodriguez-Monge, L., Taylor, R., van de Streek, J. & Wood, P. A. (2008). *J. Appl. Cryst.* **41**, 466–470.

[bb17] Nielsen, F. H. (1980). *J. Nutr.* **110**, 965–973. 10.1093/jn/110.5.965 7373441

[bb18] Pantazis, D. A., Chen, X. Y., Landis, C. R. & Neese, F. (2008). *J. Chem. Theory Comput.* **4**, 908–915. 10.1021/ct800047t 26621232

[bb19] Poellot, R. A., Shuler, T. R., Uthus, E. O. & Nielsen, F. H. (1990). *Proc. Natl Acad. Sci. USA*, **44**, 80–97.

[bb20] Rajendiran, V., Karthik, R., Palaniandavar, M., Stoeckli-Evans, H., Periasamy, V. S., Akbarsha, M. A., Srinag, B. S. & Krishnamurthy, H. (2007). *Inorg. Chem.* **46**, 8208–8221. 10.1021/ic700755p 17784750

[bb21] Sammes, P. G. & Yahioglu, G. (1994). *Chem. Soc. Rev.* **23**, 327–350.

[bb22] Samnani, P. B., Bhattacharya, P. K., Ganeshpure, P. A., Koshy, V. J. & Satish, N. (1996). *J. Mol. Catal. A Chem.* **110**, 89–94.

[bb23] Sheldrick, G. M. (2008). *Acta Cryst.* A**64**, 112–122. 10.1107/S0108767307043930 18156677

[bb24] Sheldrick, G. M. (2015*a*). *Acta Cryst.* A**71**, 3–8.

[bb25] Sheldrick, G. M. (2015*b*). *Acta Cryst.* C**71**, 3–8.

[bb26] Skoulika, S., Michaelides, A. & Aubry, A. (1995). *Acta Cryst.* C**51**, 843–846.

[bb27] Spek, A. L. (2009). *Acta Cryst.* D**65**, 148–155. 10.1107/S090744490804362X PMC2631630 19171970

[bb28] Weigend, F. & Ahlrichs, R. (2005). *Phys. Chem. Chem. Phys.* **7**, 3297–3305. 10.1039/b508541a 16240044

[bb29] Wörl, S., Fritsky, I. O., Hellwinkel, D., Pritzkow, H. & Krämer, R. (2005*b*). *Eur. J. Inorg. Chem.* pp. 759–765.

[bb30] Wörl, S., Pritzkow, H., Fritsky, I. O. & Krämer, R. (2005*a*). *Dalton Trans.* pp. 27–29. 10.1039/b417053a 15605143

[bb31] Wüllen, C. (1998). *J. Chem. Phys.* **109**, 392–399.

[bb32] Yilmaz, V. T., Hamamci, S. & Gumus, S. (2006). *Chem. Phys. Lett.* **425**, 361–366.

